# Distinctive mental health profiles in the working population: A nation-wide study from Finland: Ari Väänänen

**DOI:** 10.1093/eurpub/ckac129.686

**Published:** 2022-10-25

**Authors:** A Väänänen, J Kausto, K Gluschkoff, L Kokkinen, S Selinheimo

**Affiliations:** 1 Finnish Institute of Occupational Health, Helsinki, Finland; 2 Faculty of Social Sciences, Tampere University, Tampere, Finland

## Abstract

**Introduction:**

Studies on mental health inequalities are usually based on limited sets of mental health indicators.

**Objectives:**

Using a large number of mental health indicators, we explored whether it is possible to identify similar hierarchical rankings regardless of mental health indicators (incl. psychotherapy) among employees representing different socio-demographic statuses, and which groups of employees have the highest mismatch between mental health symptom and treatment.

**Methods:**

Employees representing different occupational classes and employees from four different areas of Finland were studied and compared. We used national register data to define psychotropic medication (purchases), sickness absence for mood disorders, and the use of psychotherapy between 2017 and 2019 and national survey data from the FinHealth 2017 Study to define the level of psychological symptoms (BDI, GHQ). We assessed the risk of each outcome by population group separately for men and women, and estimated the mismatch between symptoms (BDI/GHQ caseness) and treatment (psychotropic drugs/therapy).

**Results:**

In all the studied groups, the prevalence of mental health indicators was mostly considerably higher among women than men. The risk of register-based mental health indicators was typically higher among lower non-manual employees. In the case of some mental health indicators, we observed significant interactions between occupation class and region. Some stark mismatches were detected between symptoms and treatment in some populations, whereas at the other end of the spectrum, the correspondence between symptoms and the mobilization of care was rather high.

**Conclusions:**

Although gender is strongly linked to mental health indicators, occupational class and region influence mental health profiles in the population. There are considerable inequalities between populations in the level of professional care associated with mental health problems.

